# An efficient *Agrobacterium*-mediated transient transformation system and its application in gene function elucidation in *Paeonia lactiflora* Pall

**DOI:** 10.3389/fpls.2022.999433

**Published:** 2022-10-06

**Authors:** Shixin Guan, Xuening Kang, Jiayuan Ge, Riwen Fei, Siyang Duan, Xiaomei Sun

**Affiliations:** ^1^ College of Forestry, Shenyang Agricultural University, Shenyang, China; ^2^ Key Laboratory of Forest Tree Genetics, Breeding and Cultivation of Liaoning Province, Shenyang Agricultural University, Shenyang, China; ^3^ College of Horticulture, Shenyang Agricultural University, Shenyang, China

**Keywords:** *Paeonia lactiflora* pall., efficient transient transformation, *Agrobacterium*-mediated, stress resistance genes, physiological indexes

## Abstract

*Paeonia lactiflora* Pall. is known as the king of herbaceous flowers with high ornamental and precious medicinal value. However, the lack of a stable genetic transformation system has greatly affected the research of gene function in *P. lactiflora.* The *Agrobacterium*-mediated transient gene expression is a powerful tool for the characterization of gene function in plants. In this study, the seedlings of *P. lactiflora* were used as the transformation receptor materials, and the efficient transient transformation system with a GUS reporter gene was successfully established by *Agrobacterium* harboring pCAMBIA1301. To optimize the system, we investigated the effects of germination time, *Agrobacterium* cell density, infection time, acetosyringone (AS) concentration, co-culture time, negative pressure intensity, Tween-20 concentration and different receptor materials on the transient transformation efficiency of *P. lactiflora.* The results showed that the highest transient transformation efficiency (93.3%) could be obtained when seedlings in 2-3 cm bud length were subjected to 12 h infection of resuspension solution comprising 1.2 OD_600_ *Agrobacterium*, 200 μM AS and 0.01% Tween-20 under 10 of negative pressure intensity followed by 3 days of co-culture in darkness condition. This method is more suitable for the study of gene function in *P. lactiflora.* Subsequently, stress resistance genes *PlGPAT*, *PlDHN2* and *PlHD-Zip* were used to verify the effectiveness of this transformation system. These results can provide critical information for identification of key genes in non-model plants, such as *P. lactiflora*, and promote the development of molecular biology research for *P. lactiflora*.

## Introduction

Herbaceous peony (*Paeonia lactiflora* Pall.) belongs to the Paeoniaceae family and is known as the ‘flower phase’. It is a famous traditional flower and has been cultivated for more than three thousand years in China (Li, 1999). *P*. *lactiflora* is widely introduced because it has medicinal and excellent ornamental value as an economic plant. *P*. *lactiflora* has strong cold resistance and can survive winter in the open field in the northern region. It is an important garden flower and is very popular. In recent years, with the increasing area of cultivation, the environmental challenges faced by *P*. *lactiflora* have become more complex. Therefore, it is important to study the functions of stress resistance-related genes in *P*. *lactiflora*. At present, the commonly used gene function research is to stably inherit the target gene in this plant to obtain transgenic plants. But for *P*. *lactiflora*, a stable genetic transformation system has not been established so far, and there are still problems in the study of tissue culture of *Paeonia* plants such as contamination, browning (Zhang et al., 2021), vitrification of explants, difficulties in redifferentiation of callus, inducing robust clumps of seedlings, and rooting sterile seedlings of *P*. *lactiflora* (Zhou and Huang, 2018). Presently, domestic and foreign studies on gene function of *P*. *lactiflora* mostly use model plants, such as tobacco and *Arabidopsis thaliana*. Thus, it is necessary to establish an *Agrobacterium*-mediated high efficiency transient transformation system suitable for studying the function of the anti-stress gene of *P*. *lactiflora*.

*Agrobacterium*-mediated transient transformation of plants is a technology that can achieve transient high-level expression of target genes in a short time (Tyurin et al., 2020), which is widely used for the gene functional verification (Vaghchhipawala et al., 2011). Compared with stable transformation, it does not rely on chromosomal integration of heterologous DNA (Zheng et al., 2012), is simpler, faster and lower cost, and the process allows the processing of large numbers of plants as well as multiple gene transient expression on a single leaf analysis (Tsubasa, 2018), making this technology a powerful tool for studying the function of plant genes without genetic transformation system (Liao et al., 2017). Currently, this technology has been successfully applied to many plants, such as *A. thaliana* (Guo et al., 2016), *Betula platyphylla* (Zheng et al., 2012), *Salix babylonica* (Ji et al., 2014), *Populus euphratica* (Takata and Eriksson, 2012), *Gossypium hirsutum* (Li et al., 2018a), *Rosa chinensis* (Lu et al., 2017), and *Anemone vitifolia* (Wang, 2014). Xie et al. (2019) first applied a Tobacco Rattle Virus (TRV)-induced gene silencing technique to the peony ‘Fengdan’. After silencing the peony endogenous gene *PoPDS*, it was found that the uppermost new leaves showed a typical photobleaching phenotype. In addition, by observing the fluorescence of green fluorescent protein (GFP) in the leaves and roots of peony inoculated with TRV-GFP, it was found that TRV can silence genes in various tissues. Bian et al. (2020) used the virus-induced gene silencing (VIGS) technique to silence the *PlDELLA* gene in *P.lactiflora* and found that *PlDELLA* has a negative effect on dormancy release and plant growth. The above findings suggest that transient transformation system can be applied in the study of gene function in *Paeonia*, but mostly by means of VIGS technique (Shu et al., 2018; Wu et al., 2020). However, the overexpression of some key genes *via Agrobacterium*-mediated transient transformation system has rarely been reported in *Paeonia*.

The cold resistance of plants is closely related to the level of phosphatidylglycerol in chloroplast membrane lipids. Glycerol-3-phosphate acyltransferase (GPAT) is the first acylesterase for synthesis of phosphatidylglycerol in biofilm (Shockey et al., 2016). Recent studies revealed that the gene has a potential role in tolerance to abiotic stress in many species, such as *Ammopiptanthus mongolicus* (Xue et al., 2019), *Oryza sativa* (Imran et al., 2021) and *Forsythia viridissima* (Li et al., 2020). Li et al. (2018b) analyzed the expression level of *PlGPAT* gene under low temperature stress and found that it plays an important role in regulating the cold resistance of *P*. *lactiflora*. Many studies have shown that the *DHN* gene is involved in a cytoprotective mechanism that maintains the stability of cell membranes under stress conditions (Klimešová et al., 2020), and abiotic stress and hormone induce the expression of this gene (Yamasaki et al., 2013). The gene’s ability to improve plant tolerance to high temperature has been confirmed in a variety of plants, such as *Capsicum annuum* (Liu, 2021), *Melilotoides ruthenica* (Shen et al., 2016) and *A. mongolicus* (Shi, 2012). Chen et al. (2017) found that the expression of *PlDHN2* gene was up-regulated after high temperature treatment, which was involved in the regulation mechanism of peony’s tolerance to high temperature stress in *P*. *lactiflora*. Moreover, Homeodomain Leucine Zipper (HD-Zip) protein is a transcription factor specific to higher plants. A large number of research data show that HD-Zip protein plays a very important role in plant growth regulation and resistance to stress (Sessa et al., 1998). Therefore, various genes of the HD-ZIP family have been isolated and cloned from numerous plants, and their functions have been studied. Examples include *Dendrobium catenatum* (Huang et al., 2021), *Zea mays* (Wang et al., 2021) and *Arachis hypogaea* (Banavath et al., 2018). The drought tolerance function of the *P*. *lactiflora PlHD-Zip* gene has been confirmed by overexpression in *A. thaliana* (Wang et al., 2010).

A large number of previous studies have shown that the transient transformation efficiency of plants is influenced by the growth state of the transformed recipient material (Chen et al., 2021), *Agrobacterium* density (Li et al., 2018a), infection time (Xia et al., 2020a), acetosyringone concentration (Liu et al., 2020), co-culture time (An et al., 2020) and some other factors. In this study, we screened each factor mentioned above and established a high-efficiency transient transformation system of *P*. *lactiflora* mediated by *Agrobacterium*. This system was used to transiently overexpress the stress resistance genes *PlGPAT*, *PlDHN2* and *PlHD-Zip* in *P. lactiflora*, and detect their effects on low temperature, high temperature and drought stress. Gene expression levels and physiological indicators verified the feasibility of the transient transformation system. The transient transformation protocol we developed is fast and efficient for the functional study of the stress resistance gene of *P*. *lactiflora.*

## Materials and methods

### Seed collection and seedling preparation of *P. lactiflora*

**Figure 1:**
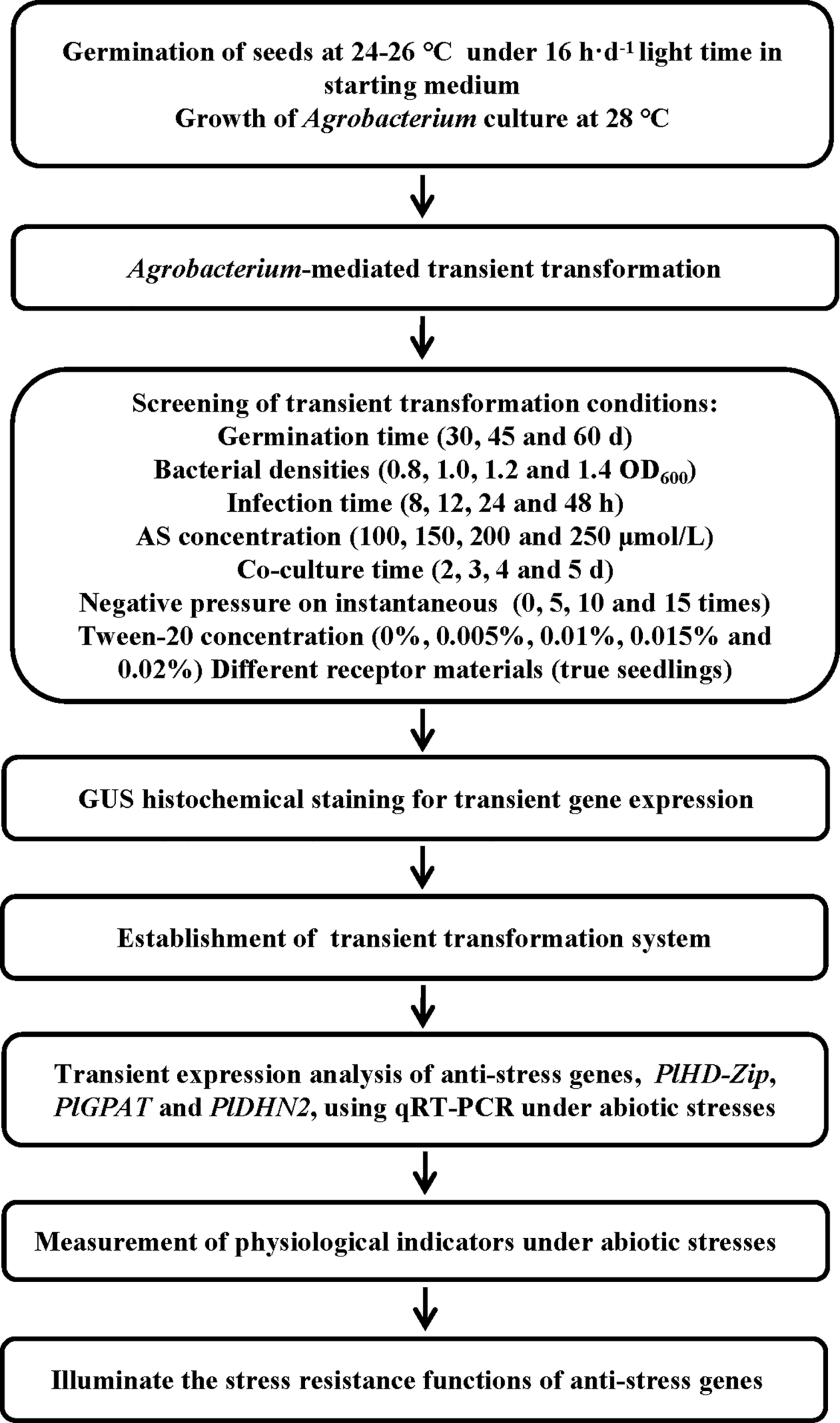
Flow chart of *Agrobacterium*-mediated transient transformation system for the development of transgenic *P. lactiflora* plants.

The aseptic tissue cultureseedlings cultivated from the herbaceous peony hybrid seeds (‘Fen Yunu’ × ‘Fen Yulou’) were collected from the Peony Germplasm Resource Garden of Shenyang Agricultural University. The pCAMBIA1301-GUS vector was gifted by Professor Yucheng Wang (Shenyang Agricultural University). The *Agrobacterium tumefaciens* EHA105 strain was purchased from Angyu Biotechnology Co., Ltd. (Angyu Biotechnology Co., Ltd., Shanghai, China). The trial flow is summarized in **Figure 1**.

**Figure 2:**
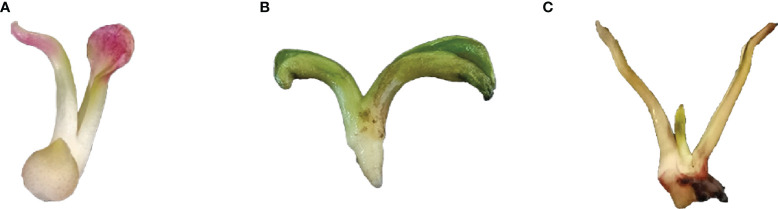
Growth States of *P. lactiflora* embryos during tissue culture. **(A-C)** Embryos at 30 **(A)**, 45 **(B)** and 60 **(C)** d after germination, respectively.

*Paeonia lactiflora* hybrid seeds were soaked in warm water for 48 h, and the seed coats were removed. The seeds were disinfected with 75% alcohol for 30s, rinsed twice with sterile water, disinfected with 0.1% HgCl_2_ solution for 5-6 min, and sterile-water rinsed 3-4 times for 1 min per time. Sterile water was then removed with sterile filter paper. The embryos were taken out and inoculated in the starting medium (MS + 0.5 mg·L^-1^ GA_3_ + 1.0 mg·L^-1^ 6-BA + 30 g·L^-1^ sucrose + 6.5 g·L^-1^ agar, pH = 5.6), and placed under the conditions of light time of 16 h·d^-1^, light intensity of 2000-3000 lx, and temperature of 24-26°C. The sterile tissue culture seedlings at 30, 45 and 60 d after germination were taken for *Agrobacterium*-mediated transient transformation (**Figure 2**).

**Figure 3:**
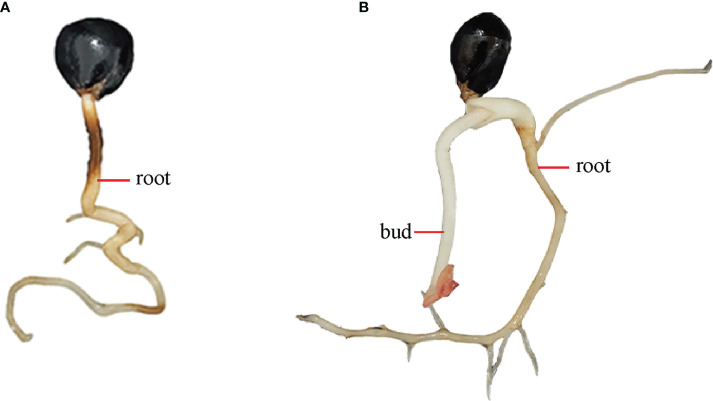
Germination states of *P. lactiflora* seed hypocotyls and epicotyls during culture. **(A)** Hypocotyl germination with the root length of 3-4 cm. **(B)** Epicotyl germination with the bud length of 2-3 cm.

The seeds were washed with washing powder for 20 min, and then flushed with water repeatedly until there is no foam. The seeds were soaked in water for 3 h, and the full seeds were retained. The seeds were disinfected with 0.5% KMnO_4_ for 40 min, and then washed with water to remove all drug residues. The seeds were soaked in 45°C warm water for 24 h until the water became turbid. The new 45°C warm water was used to continue soaking for 24 h. Next, the seeds were washed, and the floating seeds were thrown away. The seeds were stored in sand at 15-20°C, and observed every 5 days. When the root length of the seeds was 3-4 cm (**Figure 3A**), they were placed in the 4°C incubator for germination. After two months, the seeds were removed from the incubator and placed in a 15-20°C environment. Approximately 3-7 days later, seedlings with 2-3 cm buds (**Figure 3B**) were taken out to compare the effects of different receptor materials on *P. lactiflora* transient transformation efficiency.

### Construction of recombinant plasmids

The EASYspin Plus polysaccharide and polyphenol complex plant RNA rapid extraction kit (Aidlab, Beijing, China) was used to extract the total RNA of *P. lactiflora* seeds, and the RNA was reversely transcribed into cDNA using the PrimeScript™ II 1st Strand cDNA synthesis kit (Takara, Beijing, China). This cDNA was used as a template, PlGPAT-F/R, PlDHN2-F/R and PlHD-Zip-F/R are upstream and downstream primers for PCR amplification. The plant expression vector pCAMBIA1301 and the target gene with added restriction sites and protected bases were doubly digested with NcoI and BglII enzymes, and the empty expression vector and target gene fragment were recovered using a gel recovery kit (Aidlab, Beijing, China). The coding sequence (CDS) of the target gene was fused in-frame with the GUS coding region in the plant expression vector pCAMBIA1301 to construct the recombinant plasmid *pCAMBIA1301:PlGPAT/PlDHN2/PlHD-Zip* (**Supplementary Figure 1**). The empty vectors were used as control. All primers used in PCR are shown in **Supplementary Table 1**.

### *Agrobacterium*-mediated transient transformation

The constructs and pCAMBIA1301 empty vector were transformed into *Agrobacterium* EHA105 by freeze-thaw method. The bacterial solution was coated on LB solid medium containing 50 mg·L^-1^ Kan and 50 mg·L^-1^ Rif and cultivated for 2-3 d at 28°C in inverted position. The single colony was inoculated to 1.5 mL of LB liquid medium containing 50 mg·L^-1^ Kan and 50 mg·L^-1^ Rif and incubated for 24 h at 28°C and 200 rpm. The 1 mL of turbid bacterial culture was taken into 100 mL of LB liquid medium containing 50 mg·L^-1^ Kan and 50 mg·L^-1^ Rif for secondary activation. The OD_600_ values of bacterial solution were incubated to 0.8, 1.0, 1.2 and 1.4, and the culture was centrifuged at 5000 rpm for 10 min. The bacterial pellets were collected and then transferred into transformation solution (MS + 1 mmol·L^-1^ MES + 2 mmol·L^-1^ MgCl_2_ + 100, 150, 200, 250 μmol·L^-1^ AS + 30 g·L^-1^ sucrose + 0, 0.005%, 0.01%, 0.015%, 0.02% Tween-20, pH=5.6) for resuspension. The *P*. *lactiflora* seedlings were placed in a syringe containing *Agrobacterium* resuspension solution. The injection port was sealed and syringe was drawn 0, 5, 10 and 15 times with 3-5 cm each time for negative pressure treatment. Afterwards, they were poured into a wide-mouth conical flask containing *Agrobacterium* resuspension solution. The *P. lactiflora* seedlings were infected at 28°C and 200 rpm for 8, 12, 24 and 48 h. After that, they were rinsed twice with MS medium. Then *P. lactiflora* tissue culture seedlings were blotted on sterile filter paper and co-cultivated in medium (MS + 0.5 mg·L^-1^ GA_3_ + 1.0 mg·L^-1^ 6-BA + 200 μmol·L^-1^ AS + 30 g·L^-1^ sucrose + 6.5 g·L^-1^ agar, pH=5.6) for 2, 3, 4 and 5 d under dark condition. For true seedlings, they were planted in sterilized sand, and the flower pot mouth was covered with perforated preservative film. Under dark conditions, the culture was carried out for 3 d.

### Screening of transient transformation conditions of *P*. *lactiflora*

When *Agrobacterium* was used to infect *P*. *lactiflora* seedlings, the transient transformation conditions were screened, including germination time (30, 45 and 60 d), bacterial densities (0.8, 1.0, 1.2 and 1.4 OD_600_), infection time (8, 12, 24 and 48 h), AS concentrations (100, 150, 200 and 250 μmol/L), co-culture time (2, 3, 4 and 5 d) and negative pressure intensities (0, 5, 10 and 15 times), Tween-20 concentrations (0%, 0.005%, 0.01%, 0.015% and 0.02%) and different receptor materials (true seedlings). In the process of experiment design, taking one factor as single variable and other factors as standard, the effects of different influencing factors on the transient transformation efficiency of *P*. *lactiflora* seedlings were observed.

### GUS histochemical staining for transient gene expression

GUS staining was performed on the co-cultured *P*. *lactiflora* seedlings with the GUS staining kit (Real-Times, Beijing, China) for 30 plants per treatment. The seedlings were put into the GUS staining solution and placed at 37°C for 24 h in the dark. Afterwards, the seedlings were decolorized with 75% alcohol, and then transferred into 95% alcohol. The 95% alcohol was replaced 2-3 times during the period until the chlorophyll was completely destained. The blue-stained plants were considered to be positive transient transgenic plants. Transformation efficiency was calculated *via* the equation: transformation efficiency (%) = (number of seedlings stained/total number of seedlings) × 100. Three biological replicates were set for each group of experiments.

### Transient expression analysis of anti-stress genes of *P*. *lactiflora*

The transient transgenic plants were placed in a constant temperature incubator and maintained at -4 or 40°C for low or high temperature stress, respectively. The transient transgenic plants were inoculated into an initial medium containing 150 mmol·L^-1^ mannitol for drought stress. Samples were collected at 0 and 2 h after each stress treatment. The obtained samples were flash-frozen using liquid nitrogen, followed by storage at -80°C until subsequent use.

The true seedlings of transient transgenic *P*. *lactiflora* treated for 0 and 2 h under different abiotic stresses were collected, and the total RNA was extracted from the transient transgenic *P*. *lactiflora* seedlings using EASYspin Plus polysaccharide and polyphenol complex plant RNA rapid extraction kit (Aidlab, Beijing, China). RT Master Mix kit (Perfect Real Time) (Takara, Beijing, China) was used to reverse-transcribe total RNA into cDNA. The StepOnePlus™ fluorescence quantifier (Takara, Beijing, China) was used in quantitative real-time PCR (qRT-PCR) analysis. The *PlActin* (GenBank accession no. JN105299) was selected as the internal reference gene (Ge et al., 2014). The reaction system contained 10 μL of 2x TB Green Premix Ex Taq™ (Tli RNaseH Plus) (Takara, Beijing, China), 0.4 μL of 50x ROX Reference Dye, 5.6 μL of ddH_2_O, 1 μL of each primer (10 μM), and 2 μL of cDNA in a final volume of 20 μL. A two-step amplification standard procedure was adopted. The amplification parameters ;were as follows: predeformation at 95°C for 30 s; 40 cycles at 95°C for 5 s and 60°C for 30 s. The dissolution curve program was carried out after 40 cycles under 60-95°C at the increment of 0.5°C/5 s. The gene relative expression levels were calculated according to the 2^-ΔΔCt^ method (Schmittgen and Livak, 2008). Each sample included three biological replicates. All primers for qRT-PCR are shown in **Supplementary Table 2**.

### Determination of physiological indicators of transient transgenic plants

The true seedlings of *P*. *lactiflora* treated with abiotic stress were collected for the determination of physiological indexes. Superoxide dismutase (SOD) and peroxidase (POD) activity assays were determined according to the method of Dhindsa et al. (1981). The activity of catalase (CAT) and the content of proline were determined following the previously described method (Li, 2000). The content of malondialdehyde (MDA) was determined according to the method of Wang et al. (2010). The measurement of soluble sugar content referred to the published method (Tang, 1999). All experiments were performed in three biological replicates and each replicate contained at least 10 transient transgenic true seedlings.

### Statistical analysis

The data were analyzed with SPSS software (Version 22.0, SPSS Inc, USA). The experiments were performed in three biological replicates and subjected to one-way analysis of variance (ANOVA) followed by Duncan’s multiple range test (DMRT) at P < 0.05 significance level.

## Results

### Effect of germination time on transient transformation efficiency of *P. lactiflora*

In order to study the effect of germination time on the transient transformation efficiency of *P. lactiflora*, tissue culture seedlings of *P. lactiflora* at 30, 45 and 60 d after germination were selected and transformed with *Agrobacterium* EHA105 containing *GUS* gene (**Supplementary Figure 2**). The cotyledons of tissue culture seedlings at 30 and 45 d after germination had blue spots, and the GUS positive tissue culture seedlings at 30 d after germination had the highest transformation rate of 33.3%. However, only one true leaf of 60-day-old tissue culture seedlings had a small area of micro-blue on the edge, indicating that GUS expression efficiency decreased with increasing number of days after germination. Therefore, in the later experiment, 30-day-old *P. lactiflora* tissue culture seedlings were selected as transformation receptor materials.

### Effect of bacterial concentration on transient transformation efficiency of *P. lactiflora*

Four concentrations of *Agrobacterium* were set in this experiment, and the OD_600_ values were 0.8, 1.0, 1.2 and 1.4, respectively (**Supplementary Figure 3**). When the OD_600_ value increased from 0.8 to 1.2, the instantaneous transformation efficiency increased gradually. When the OD_600_ value was 1.2, the instantaneous transformation efficiency was the highest with the transformation rate of 33.3%. When the OD_600_ value increased to 1.4, the number of dead *Agrobacterium* cells increased, and the instantaneous transformation rate decreased to 30%. Therefore, in this experiment, the optimal bacterial concentration for *P. lactiflora* transient transformation system was 1.2.

### Effect of infection time on transient transformation efficiency of *P. lactiflora*

In the study of the effect of infection time on the instantaneous transformation efficiency of *P. lactiflora*, we found that the instantaneous transformation efficiency of *P. lactiflora* was 26.7% with 8 h of infection period. With the increase of infection time, the instantaneous transformation efficiency increased. The highest instantaneous transformation efficiency was obtained with 24 h of infection period, and the transformation rate reached 40%. When the tissue culture seedlings were infected for 48 h, the instantaneous transformation efficiency was the lowest (**Supplementary Figure 4**). The tissue culture seedlings of *P. lactiflora* appeared softening and wilting, and the state of tissue culture seedlings was still unable to recover after co-culture, which was unable to be used for subsequent research.

### Effect of AS concentration on transient transformation efficiency of *P. lactiflora*

Appropriate addition of AS can improve the transformation ability of *Agrobacterium* (Zou, 2011). In this study, four concentrations of AS were set as 100 μmol·L ^−1^, 150 μmol·L ^−1^, 200 μmol·L ^−1^ and 250 μmol·L^−1^. Adding appropriate amount of AS in the transformation solution could improve the instantaneous transformation efficiency of *P. lactiflora*. With the increase of AS concentration, the instantaneous transformation rate of *P. lactiflora* increased. Adding 200 μmol·L^−1^ AS to the transformation solution, the number of successfully dyed tissue culture seedlings was the largest, and the transformation rate reached 40%. When the AS concentration increased to 250 μmol·L^-1^, the instantaneous transformation rate decreased to 36% (**Supplementary Figure 5**). Therefore, the optimal AS concentration in the transient transformation system of *P. lactiflora* was 200 μmol·L^−1^.

### Effect of co-culture time on transient transformation efficiency of *P. lactiflora*

Four gradients of co-culture time were set in this experiment. When the co-culture time was 2 d, the instantaneous transformation efficiency was the lowest, and the transformation rate was 17.6%. When the co-culture time increased to 3 d, the instantaneous transformation rate reached the highest value of 40% (**Supplementary Figure 6**). Due to the absence of antibiotics in the co-culture medium, the overgrowth of *Agrobacterium* led to browning and softening of tissue culture seedlings with the increase of co-culture time, which was consistent with the low transformation efficiency observed.

### Effect of negative pressure on instantaneous transformation efficiency of *P. lactiflora*

External application of appropriate negative pressure treatment can form small wounds on the surface of plant tissue, which is helpful for the infiltration of *Agrobacterium* (Mo et al., 2019). Four different negative pressure intensities (0, 5, 10, 15) were set in this experiment. When the negative pressure treatment was not carried out, the instantaneous transformation rate of *P. lactiflora* was 40%. When the negative pressure intensity was 5, the instantaneous transformation efficiency was 50%. When the negative pressure intensity increased to 10, the instantaneous transformation efficiency of *P. lactiflora* was the highest, reaching 63.3%. This was 1.58 times higher than that of no negative pressure treatment (**Supplementary Figure 7**). When the negative pressure intensity was 15, the tissue culture seedlings were obviously injured, and softening and wilting occurred. It was unable to carry out subsequent experiments. Therefore, the optimum negative pressure strength for *P. lactiflora* transient transformation system was 10.

### Effect of Tween-20 concentration on the instantaneous transformation efficiency of *P. lactiflora*

Tween-20 is a commonly used surfactant, and an appropriate amount of Tween-20 can help *Agrobacterium* infiltrate into the intercellular space of plant cells, thereby improving the transient transformation efficiency (Zhong et al., 2016). In this study, five gradient concentrations of Tween-20 were set in the process of transient transformation for tissue culture seedlings. There was no difference in the instantaneous transformation efficiency between 0.005% and 0% Tween-20. When the concentration of Tween-20 increased to 0.01%, the instantaneous transformation efficiency increased significantly, and the transformation rate reached 73.3%. This was 1.08 times higher than that of no Tween-20. However, when the concentration of Tween-20 increased to 0.02%, the instantaneous transformation efficiency decreased to 57% (**Supplementary Figure 8**). The results showed that 0.01% Tween-20 was the optimal concentration for transient transformation.

### Effect of different receptor materials on transient transformation efficiency of *P. lactiflora*

In this study, the true seedlings with buds of 2-3 cm were used as the receptor materials for transient transformation of *P. lactiflora*. It was found that when the seedlings were transformed under the optimal transient transformation conditions selected in the previous study, the seedlings showed softening and wilting after 24 h of *Agrobacterium* suspension infection, indicating that the *P. lactiflora* seedlings were invaded by *Agrobacterium*. This was not conducive to the later experiment. Therefore, the infection time of 8 h, 12 h and 24 h were set in this experiment. The instantaneous transformation rate was 63.3% when the seedlings were infected in the *Agrobacterium* suspension for 8 h. Then, the instantaneous transformation rate increased first and then decreased with the increase of infection time. The instantaneous transformation rate was the highest at 12 h, reaching 93.3%. This experiment suggested that the optimal infection time was 12 h when the *P. lactiflora* seedling was used as transformation receptor material (**Supplementary Figure 9**).

### Identification of transient transgenic *P. lactiflora*

**Figure 4:**
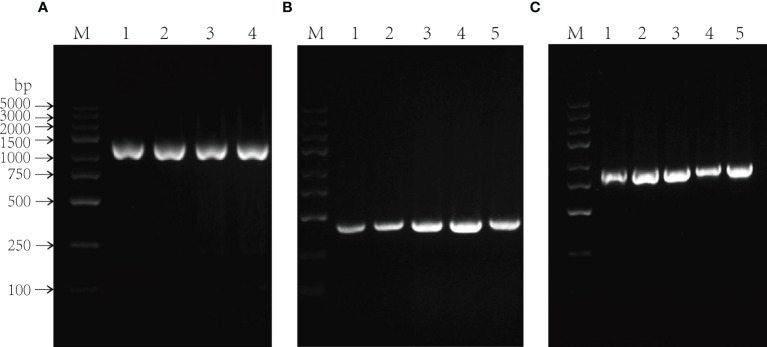
Molecular analysis of *Agrobacterium tumefaciens* containing recombinant expression vector. **(A-C)** PCR analysis of *Agrobacterium tumefaciens* using *PlGPAT* **(A)**, *PlDHN2* **(B)** and *PlHD-Zip* **(C)** gene-specific primers, respectively. PCR products in 1347, 399 and 972 bp were observed in each *Agrobacterium tumefaciens* using *PlGPAT*, *PlDHN2* and *PlHD-Zip* gene-specific primers, respectively. M, DL2000 marker; 1-5, different *Agrobacterium tumefaciens*.

The constructs *pCAMBIA1301:PlGPAT/PlDHN2/PlHD-Zip* were transformed into *Agrobacterium* EHA105 by freeze-thaw method, respectively. The single colonies were selected for oscillation culture. The shaken *Agrobacterium* solution was subjected to bacterial solution PCR. The products of 1347, 399 and 972 bp were amplified from the three *Agrobacterium* solutions with different recombinant vectors, respectively. The products were all in line with the expected size, indicating functional recombinant vectors *pCAMBIA1301:PlGPAT/PlDHN2/PlHD-Zip* and successful *Agrobacterium* transformations (**Figure 4**).

**Figure 5:**
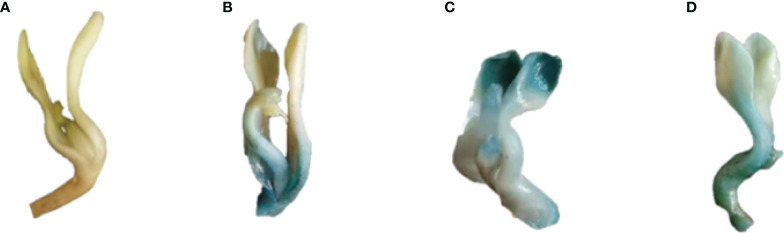
GUS staining of transient transgenic *P. lactiflora* seedlings. **(A)** Seedlings as control exhibit no GUS expression. **(B-D)** Seedlings with transient expression of *PlGPAT* **(B)**, *PlDHN2* **(C)** and *PlHD-Zip* **(D)** gene exhibit GUS expression, respectively.

Subsequently, the recombinant over-expression vector containing stress-resistant gene of *P. lactiflora* was transiently transformed into *P. lactiflora* true seedlings by *Agrobacterium*-mediated method, and the pCAMBIA1301 empty vector containing *GUS* reporter gene was transiently transformed into *P. lactiflora* seedlings as the blank control group. The transient transgenic *P. lactiflora* true seedlings were immersed in GUS staining solution (**Figure 5**). The transient transformation of *PlGPAT*, *PlDHN2* and *PlHD-Zip* genes in *P. lactiflora* seedlings elicited blue spots visible to naked eyes, indicating that the transient transformation system of *P. lactiflora* was successfully used to transiently transform the three stress-resistant genes into *P. lactiflora* seedlings.

### Transient overexpression of stress-resistance gene in *P. lactiflora*

**Figure 6:**
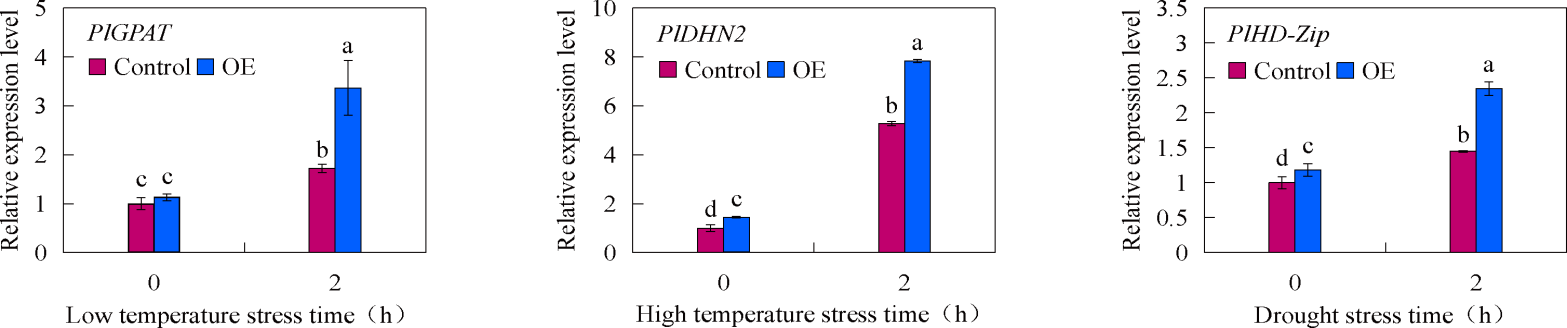
Transient expression analysis of *P. lactiflora* stress resistance genes under abiotic stresses. Error bars indicate standard deviation of three replicates. Different letters mean significantly different by Duncan’s multiple range test at *P* < 0.05.

In order to verify the feasibility of the established transient transformation system, the overexpression vectors of *PlGPAT*, *PlDHN2* and *PlHD-Zip* were constructed in this study. The established transient transformation system was used to transiently transformed these constructs into *P. lactiflora* true seedlings, and the expression changes of three stress-resistant genes under abiotic stress were detected by qRT-PCR method (**Figure 6**). The results showed that there was no significant difference in the expression levels of stress-resistant genes between the control and transient transgenic seedlings under normal growth condition. After abiotic stress treatment, the expression levels of stress-resistant genes in the control and transient transgenic seedlings were significantly increased, and the expression levels of stress-resistant genes in the transient transgenic seedlings were significantly higher than those in the control seedlings. This indicated that *PlGPAT*, *PlDHN2* and *PlHD-Zip* were successfully transiently overexpressed in *P. lactiflora* seedlings and are responsible for stress resistance functions.

### Changes in physiological indexes of transient over-expression *P. lactiflora*

**Figure 7:**
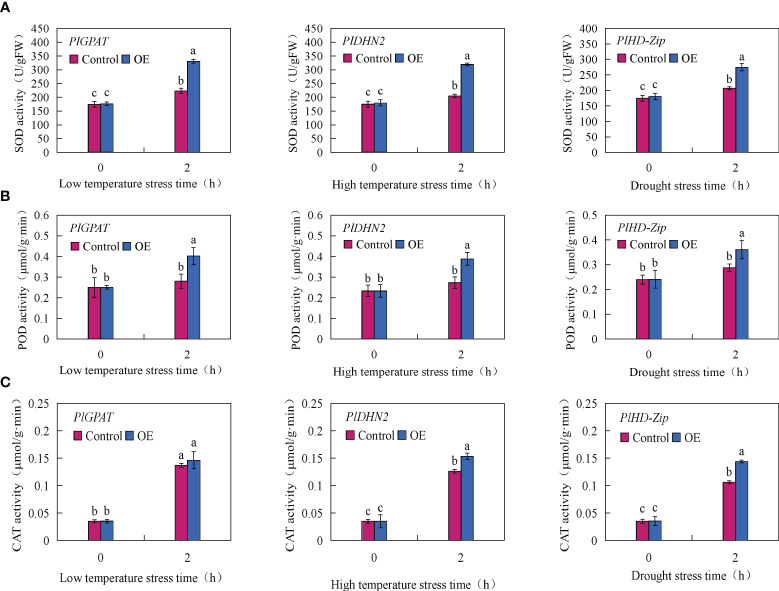
Analysis of antioxidant enzyme activity in *P. lactiflora* seedlings with transient overexpression of stress resistance genes. **(A)** SOD activity. **(B)** POD activity. **(C)** CAT activity. Error bars indicate standard deviation of three replicates. Different letters mean significantly different by Duncan’s multiple range test at *P* < 0.05.

**Figure 8:**
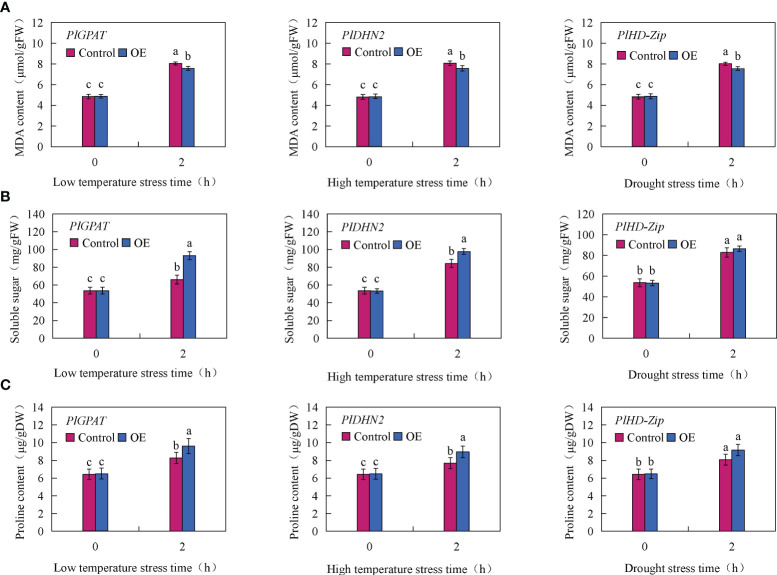
Analysis of Physiological index in *P. lactiflora* seedlings with transient overexpression of stress resistance genes. **(A)** MDA content. **(B)** soluble sugar content. **(C)** Proline content. Error bars indicate standard deviation of three replicates. Different letters mean significantly different by Duncan’s multiple range test at *P* < 0.05.

To further verify the function of stress resistance genes *PlGPAT*, *PlDHN2* and *PlHD-Zip*, the activities of three major antioxidant enzymes and the contents of MDA, soluble sugar and proline in transient transgenic true seedlings were measured. The results of antioxidant enzyme activities showed that there was no significant difference in SOD, POD and CAT activities between the control and transient transgenic *PlGPAT P. lactiflora* tissue culture seedlings under normal growth condition. After low temperature stress treatment, the activities of SOD, POD and CAT in *P. lactiflora* seedlings were significantly increased, and the activities of SOD, POD and CAT in transient transgenic seedlings were significantly higher than those in control seedlings. Under high temperature or drought stress, the same results were also obtained for the analysis of antioxidant enzyme activities in the seedlings of transiently transformed *PlDHN2* and *PlHD-Zip* (**Figure 7**). The analysis of cell membrane permeability physiological indexes showed that under normal growth conditions, there was no significant difference in MDA content between the control and transient transgenic seedlings of *P*. l*actiflora*. After abiotic stress, the MDA contents of the control and transient transgenic seedlings containing *PlGPAT*/*PlDHN2*/*PlHD-Zip* gene increased, and the content of transient transgenic seedling was significantly lower than that of the control seedling (**Figure 8A**). The content analysis of organic osmolytes showed that the soluble sugar and proline contents of the control and transient transgenic peony seedlings were significantly increased compared with the normal growth condition after abiotic stress treatment, and the soluble sugar and proline contents of the transient transgenic seedlings were significantly higher than those of the control seedlings (**Figures 8B**, **C**). The above research results demonstrated that the transient transformation system of *P. lactiflora* was successfully used to transfer the stress-resistant gene of *P. lactiflora* into the seedlings of *P. lactiflora* and exert its stress-resistant function, indicating that the transient transformation system of *P. lactiflora* established in this study could be used to study the function of the stress-resistant gene in *P. lactiflora*.

## Discussion

The *Agrobacterium*-mediated transient transformation of plants is mainly involved in injection and infiltration method. In the study, these two methods were applied. With injection method, due to the thickness of leaves and tightness of cell tissues of *P*. *lactiflora* tissue culture seedlings, the bacterial solution could not be diffused after the injection of *Agrobacterium* suspension. This resulted in a lower instantaneous transformation efficiency. In contrast, with the infiltration method, the instantaneous transformation was completed after only a few hours of submerging of whole *P*. *lactiflora* seedlings in *Agrobacterium* tumefaciens resuspension solution. This result suggests that *Agrobacterium* infiltration method is more suitable for transient transformation of *P*. *lactiflora*.

*Agrobacterium*-mediated transient transformation is a complex process. In order to obtain high transformation rate with high expression of target genes in the tissue culture seedlings of *P*. *lactiflora*, we screened the optimal transformation conditions. Firstly, the growth status of transformation receptor materials has a dramatic impact on the infection of *Agrobacterium*. After a long time of germination, seedlings had thicker cotyledons and tighter cell tissues, which could hinder the infection of *Agrobacterium*. Seedlings at 30 days after germination had tender cotyledons, which was beneficial for the infection of *Agrobacterium* and had the highest transient transformation efficiency. Wu (2015) found that fresh and tender leaves were more conducive to the improvement of transient expression efficiency than old leaves in mulberry. Takata and Eriksson. (2012) also considered that the transformation rate of young leaves was higher than that of old leaves in the establishment of transient transformation system of hybrid poplar.

Previous studies have shown that the *Agrobacterium* density and infection time directly determine the instantaneous transformation efficiency of plants (Mi et al., 2009). It is necessary to ensure that sufficient *Agrobacterium* infiltrates into plant cells without damaging the plant. In this study, we screened the concentration of bacterial solution and infection time. When the tissue culture seedlings of *P. lactiflora* were immersed in the *Agrobacterium* suspension with OD_600_ value of 1.2 for 24 h, the final number of GUS positive plants was the highest, with less toxicity in the tissue culture seedlings of *P. lactiflora*. In all previous works establishing transient transformation systems for *Malus sieversii* (Li et al., 2018c), *Pinus tabulaeformis* (Liu et al., 2020) and *Iris foetidissima* (An et al., 2020) all screened the bacterial solution concentration and infestation time were screened. The results suggested that too low a bacterial solution concentration or too short an infestation time would make the infestation ineffective and lead to plant damage. In addition, it has been reported that many proteins encoded by *Vir* gene play a crucial role in *Agrobacterium*-mediated transformation, and the activity of *Vir* gene carried by Ti plasmid plays a critical role for the infection ability of *Agrobacterium* (Gelvin, 2003). *Vir* gene expression is induced by phenolic compounds, and appropriate accumulation of them can promote the integration of target genes to improve the transient transformation efficiency (Zou, 2011). AS is one of the most commonly used active phenolic compounds, which is a natural signal molecule produced by damaged plant cells. It can expand the host range of some rhizobium strains (Zheng et al., 2012). Faizal and Geelen. (2012) found that the addition of 100 μmol·L^-1^ AS in *Agrobacterium* suspension could make the transient transformation efficiency of *Maesa lanceolata* reach the highest level. This concentration was also suitable for the transient transformation of the whole bamboo (Chen et al., 2021) and *I. foetidissima* leaves (An et al., 2020). Li et al. (2018a) used three different AS concentrations in the transient transformation assay of upland cotton seedlings, and found that GUS staining and activity were the highest when the AS concentration is at 165 μmol·L^-1^. It can be seen that the optimal AS concentrations for different plants are not the same. In this study, we found that the addition of AS with a concentration of 200 μmol·L^-1^ in the *Agrobacterium* suspension could achieve the highest transient transformation efficiency for *P. lactiflora*. This is consistent with the optimal AS concentration in the transient transformation system for *Salsola laricifolia* (Xia et al., 2020a).

It takes a certain time for *Agrobacterium* to transfer the target gene into plant cells. The transformation of plants by *Agrobacterium* will not complete if co-culture time is too short. On the other hand, it will cause excessive reproduction of *Agrobacterium* and irreversible damage to plants if co-culture time is too long, which reduces the instantaneous transformation rate. An et al. (2020) found that the best transient co-culture time of *I. foetidissima* leaves was 4 days. Both Faizal and Geelen (2012) and Li et al. (2018a) considered that the 5 days after infection of *M. lanceolata* and *G. hirsutum* was the best co-culture time for gene function research. In this experiment, since no antibiotic was added to the co-culture medium, the excessive growth of *Agrobacterium* led to browning and death of tissue culture seedlings of *P. lactiflora* with the extension of co-culture time. This was consistent with the results obtained by previous researchers (Zhuo et al., 2019). Therefore, we believed that 3 days was the optimal co-culture time for the transient transformation in *P. lactiflora*.

A previous study reported that external treatments, such as negative pressure, cotyledon puncture, and surfactant application, could significantly improve plant transient transformation efficiency (Xia et al., 2020b). In this study, sterile needles were used to scratch the cotyledon surface of *P*. *lactiflora* tissue culture seedlings for cotyledon puncture treatment. It was found that the transformed *P*. *lactiflora* tissue culture seedlings blackened due to the large scratch area, which could not meet the requirements of subsequent experiments. The negative pressure treatment was carried out on the tissue culture seedlings of *P*. *lactiflora*. The results showed that the treatment with intensity of 10 could significantly improve the instantaneous transformation efficiency of *P*. *lactiflora*. This is because the negative pressure treatment could form a slight wound on the surface of the tissue culture seedlings of *P*. *lactiflora*, which promoted the infection of *Agrobacterium* without harming plants. In the transient transformation assays of *Diospyros kaki* Thunb. (Mo et al., 2019), *Medicago sativa* (Wu et al., 2021) and *Caladium bicolosr* (Li, 2018), it was also found that the transient expression rate of the reporter gene was significantly increased after negative pressure treatment. Surfactants significantly decrease the surface tension by adsorption between the gas-liquid or liquid-liquid interface, This increases the permeability of plant cells and promote the transformation of *Agrobacterium*. Triton X-100, Tween-20 and Silwet L-77 are commonly used surfactants in the transient transformation process. Liu et al. (2019) found that Silwet L-77, Tween-20 and Triton X-100 all improved the transient transformation efficiency of *Caragana intermedia* to some extent. Among them, a small amount of Tween-20 can significantly improve the permeability of plant cells (Zhong et al., 2016). It helps more *Agrobacterium* enter the plant cell gap and hence improves the efficiency of transient transformation (Kalpana et al., 2016). In our study, we found that adding 0.01% Tween-20 in the *Agrobacterium* suspension could make the instantaneous transformation rate of peony reach the highest level. Mi et al. (2009) found that both 0.01% Triton X-100 and 0.01% Tween-20 could improve the transient expression efficiency of *A. thaliana*, and 0.01% Tween-20 had a better effect. Similar conclusions have been reached in the transient transformation systems of many herbaceous plants, such as *B. platyphylla*, *P. euphratica*, *Tamarindus indica*, *Duboisia myoporoides* and *S. babylonica* (Ji et al., 2014).

The culture process of *P*. *lactiflora* tissue culture seedlings is relatively complex, and the explants are prone to contamination. However, the culture of *P. lactiflora* true seedlings is relatively simple, and the contamination rate is low. The seeds used for the cultivation of *P. lactiflora* true seedlings can be stored in a large amount in sand, ensuring the sufficient transformation materials. In this experiment, the effects of different receptor materials on the transient transformation efficiency of *P. lactiflora* were studied on the basis of the optimal transient transformation conditions obtained by screening the tissue culture seedlings of *P. lactiflora*. The results showed that the optimal infection time was 12 h, and the transient transformation rate could reach 93.3%. Compared with tissue culture seedlings as transformation receptor materials, the instantaneous transformation operation of true seedlings was more convenient. It does not need to be carried out on ultra-clean bench, and the instantaneous transformation rate is higher. The result suggested that the transient transformation system with *P. lactiflora* true seedling as receptor material was more suitable for *P. lactiflora*.

The transient expression of plants has been widely used in gene function verification. For plants with no mature genetic transformation system such as *P. lactiflora*, it is very difficult to study the gene function associated with obvious phenotype. Therefore, it is necessary to establish transient transformation system for gene function exploration (Li et al., 2020). Currently, transient transformation systems have been widely used to study gene functions in plants. Zang et al. (2015) overexpressed the *ThZFP1* gene in *Tamarix hispida* through an *Agrobacterium*-mediated transient transformation system and verified its positive regulation in proline accumulation, as well as SOD and POD activities under salt and osmotic stress conditions. Using a transient transformation system (Liu et al., 2019), it was also found that transient expression of the *CiDREB1C* gene enhanced tolerance to drought and salt stress and reduced sensitivity to ABA in *C. intermedia* using a transient transformation system (Liu et al., 2019). Liao et al. (2021) conducted transient overexpression and gene silencing of *SgCPR1* and *SgCPR2*, and found that *SgCPR1* and *SgCPR2* played a particularly important role in the biosynthesis of mogroside in *Siraitia grosvenorii*. The system can not only overexpress endogenous genes of plants, but also transiently transform exogenous genes of other plants. For this reason, the gene function can be studied more widely. Moreover, the *Agrobacterium*-mediated transient transformation system can also be applied to promoter, transcription factor, protein subcellular localization and interaction analysis (Takata and Eriksson, 2012; Liu et al., 2020). Therefore, the establishment of *Agrobacterium*-mediated transient transformation system of *P*. *lactiflora* is of great research significance.

In this study, the transient overexpression of *P*. *lactiflora* resistance genes *PlGPAT*, *PlDHN2* and *PlHD-Zip* were carried out using the established transient transformation system in *P. lactiflora*. qRT-PCR analysis showed that the expression levels of stress-resistant genes in transgenic plants increased after abiotic stress treatment, which was significantly higher than those in control plants after abiotic stress treatment. The results of physiological index measurements showed that the proline and soluble sugar content as well as SOD, POD and CAT activity in the transiently overexpressed *P*. *lactiflora* resistance gene plants were significantly higher than those in control plants after abiotic stress treatment, and the MDA level was lower than that in control plants. It can be concluded that the stress resistance genes *PlGPAT*, *PlDHN2* and *PlHD-Zip* can be successfully transferred into the *P*. *lactiflora* seedlings using transient transformation system, and have stress resistance functions under abiotic stresses.

## Conclusion

The present study established an efficient *Agrobacterium*-mediated transient transformation system for *P*. *lactiflora*. The established transient transformation system was used to achieve transient overexpression of *P*. *lactiflora* resistance genes in a relatively short period of time, and the stress resistance indicators of transgenic plants were detected. The results showed that all three resistance genes served to enhance the resistance of *P*. *lactiflora* to abiotic stress treatment. The transient transformation system we developed can be successfully applied for gene transient overexpression and function analysis in *P*. *lactiflora*. Consequently, the transient transformation system will promote functional analyses of *P*. *lactiflora* stress-responsive genes.

## Data availability statement

The datasets presented in this study can be found in online repositories. The names of the repository/repositories and accession number(s) can be found below: https://www.ncbi.nlm.nih.gov/genbank/, JN105299.1, https://www.ncbi.nlm.nih.gov/genbank/, KJ914575.1, https://www.ncbi.nlm.nih.gov/genbank/, KY272747.1, https://www.ncbi.nlm.nih.gov/genbank/, MN832790.1.

## Author contributions

XS, XK and JG designed the project. XK and JG completed the tissue culture and seedling culture of *P*. *lactiflora.* XK, RF and SD completed the screening experiment of transient transformation conditions of *P*. *lactiflora* mediated by *Agrobacterium*. JG and SG participated in GUS staining and decolorization test. XK and RF completed the cloning of stress related genes and construction of recombinant expression vector of *P*. *lactiflora.* This article was written by SG, XK and JG. All authors contributed to the article and approved the submitted version.

## Funding

This work was supported by the National Natural Science Foundation of China (32071814 and 31470696).

## Conflict of interest

The authors declare that the research was conducted in the absence of any commercial or financial relationships that could be construed as a potential conflict of interest.

## Publisher’s note

All claims expressed in this article are solely those of the authors and do not necessarily represent those of their affiliated organizations, or those of the publisher, the editors and the reviewers. Any product that may be evaluated in this article, or claim that may be made by its manufacturer, is not guaranteed or endorsed by the publisher.
