# Supplementary material for: An efficient Agrobacterium-mediated transient transformation system and its application in gene function elucidation in Paeonia lactiflora Pall
Source: Front Plant Sci. 2022 Oct 6;13:999433. doi: 10.3389/fpls.2022.999433 (PMC9582852; doi:10.3389/fpls.2022.999433)
Supplement: Supplementary file 1 [file DataSheet_1.docx]

Supplementary Material

# Supplementary Figures and Tables

## Supplementary Figures


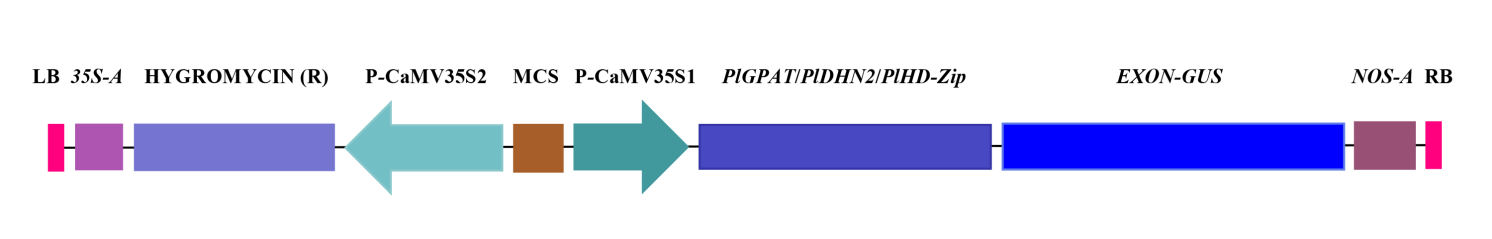


**Supplementary Figure 1.** T-DNA regions of binary vector pCAMBIA1301 applied for the transient transformation.


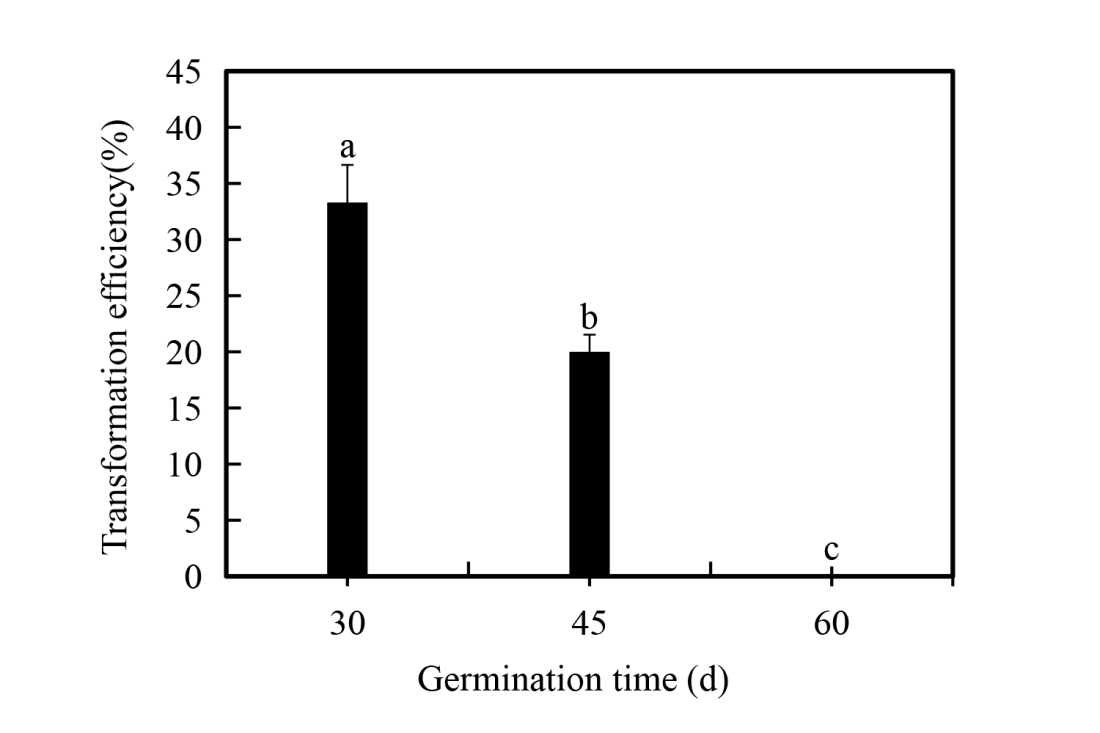


**Supplementary Figure 2.** Effects of germination time on transient transformation rate of *P. lactiflora*. Overnight culture of *Agrobacterium* (1.2 OD_600_) was re-suspended in transformation solution (MS + 1 mmol·L^-1^ MES + 2 mmol·L^-1^ MgCl_2_ +200 μmol·L^-1^ AS + 30 g·L^-1^ sucrose, pH=5.6). The infection time was 12 h, and co-culture time was 3 d. Error bars indicate standard deviation of three replicates. Different letters mean significantly different by Duncan’s multiple range test at *P* < 0.05.


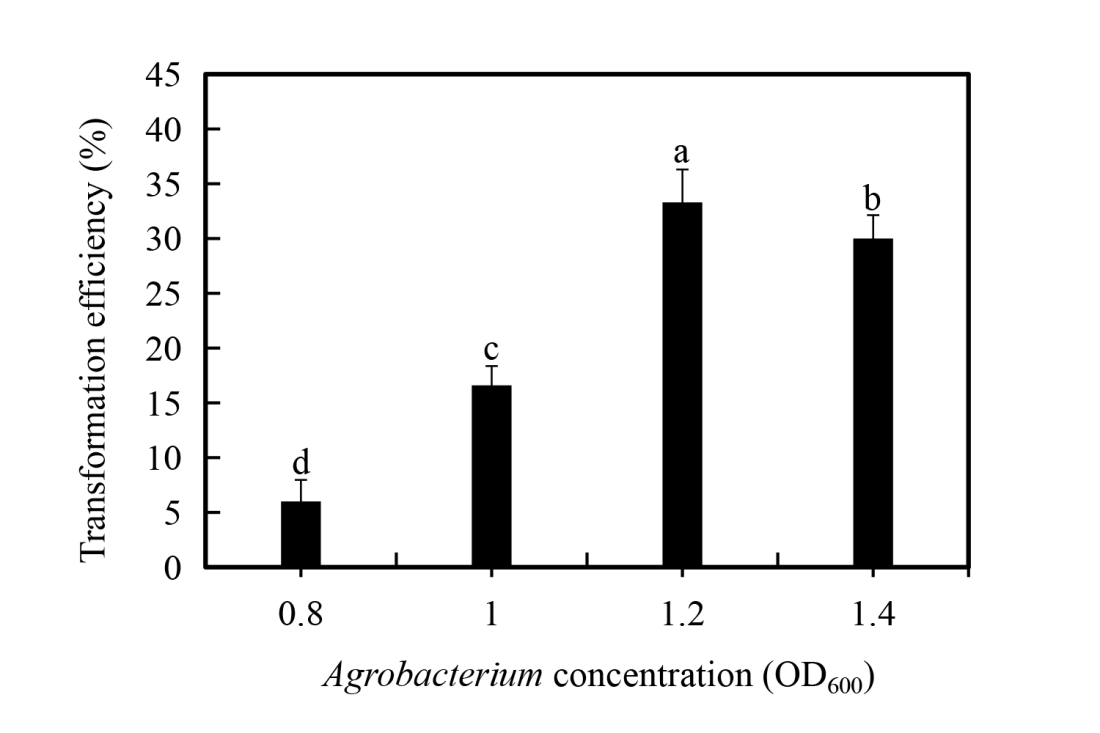


**Supplementary Figure 3.** Effects of *Agrobacterium* density on transient transformation rate of *P. lactiflora*. Overnight culture of *Agrobacterium* was re-suspended in transformation solution (MS + 1 mmol·L^-1^ MES + 2 mmol·L^-1^ MgCl_2_ +200 μmol·L^-1^ AS + 30 g·L^-1^ sucrose, pH=5.6). The germination time was 30, infection time was 12 h, and co-culture time was 3 d. Error bars indicate standard deviation of three replicates. Different letters mean significantly different by Duncan’s multiple range test at *P* < 0.05.


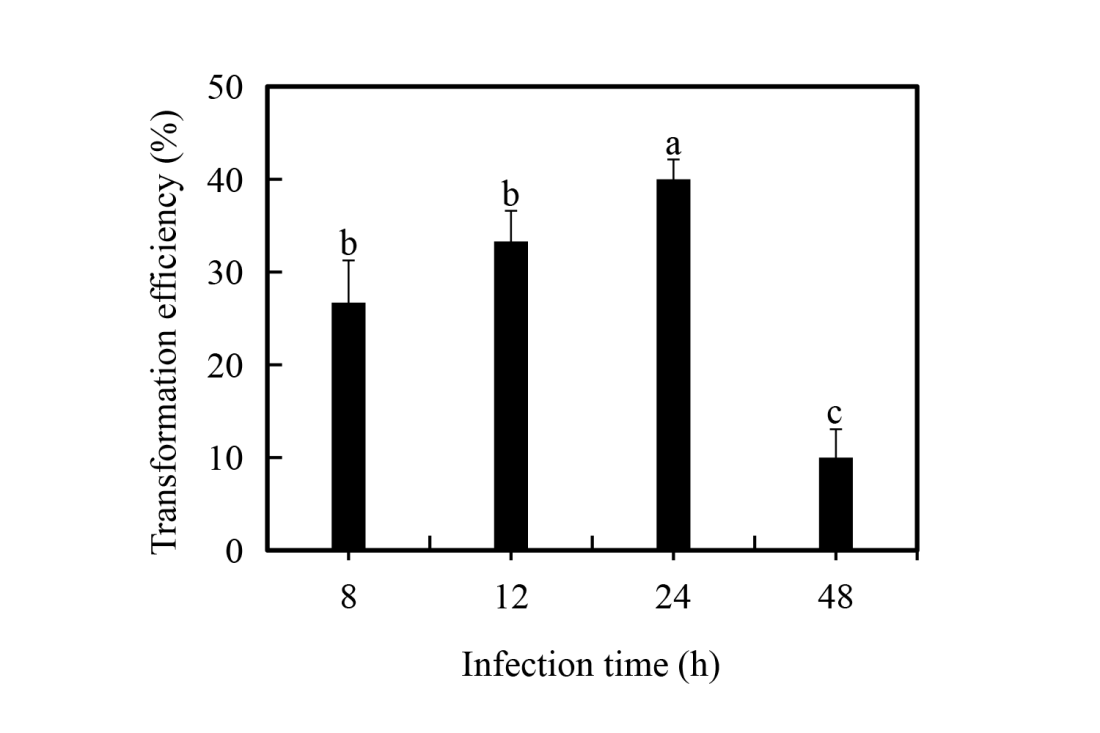


**Supplementary Figure 4.** Effects of infection time on transient transformation rate of *P. lactiflora*. Overnight culture of *Agrobacterium* (1.2 OD_600_) was re-suspended in transformation solution (MS + 1 mmol·L^-1^ MES + 2 mmol·L^-1^ MgCl_2_ +200 μmol·L^-1^ AS + 30 g·L^-1^ sucrose, pH=5.6). The germination time was 30, and co-culture time was 3 d. Error bars indicate standard deviation of three replicates. Different letters mean significantly different by Duncan’s multiple range test at *P* < 0.05.


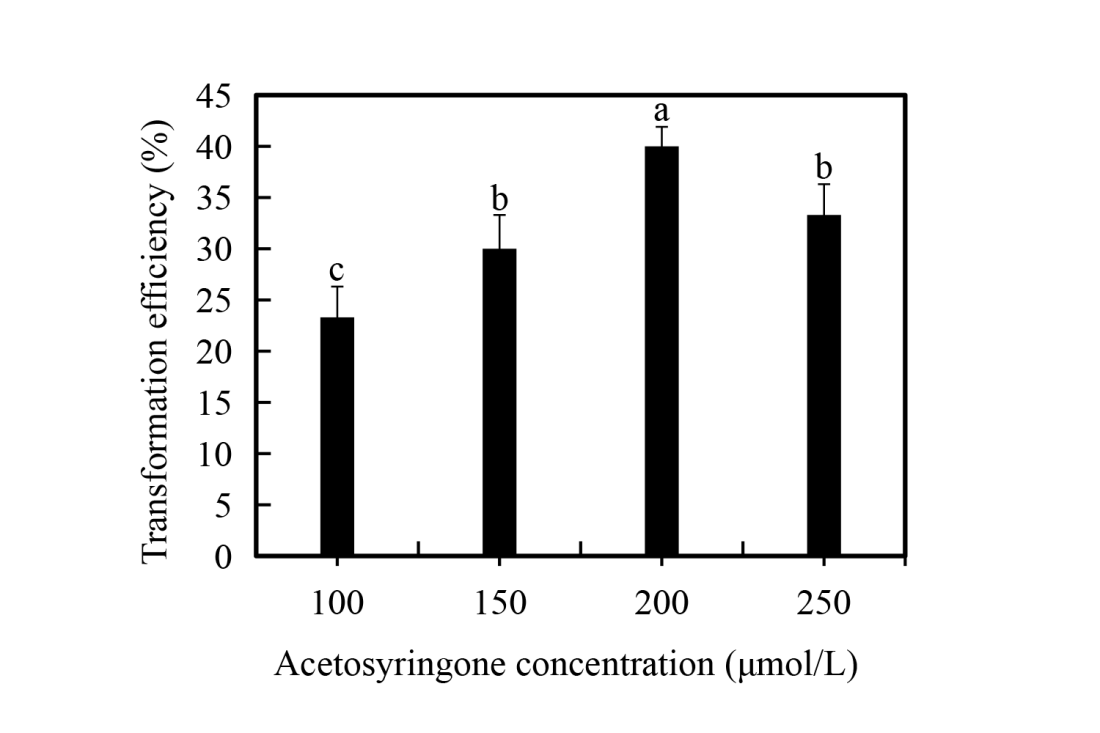


**Supplementary Figure 5.** Effects of acetosyringone concentration on transient transformation rate of *P. lactiflora.* Overnight culture of *Agrobacterium* (1.2 OD_600_) was re-suspended in transformation solution (MS + 1 mmol·L^-1^ MES + 2 mmol·L^-1^ MgCl_2_ + 30 g·L^-1^ sucrose, pH=5.6). The germination time was 30, infection time was 24 h, and co-culture time was 3 d. Error bars indicate standard deviation of three replicates. Different letters mean significantly different by Duncan’s multiple range test at *P* < 0.05.


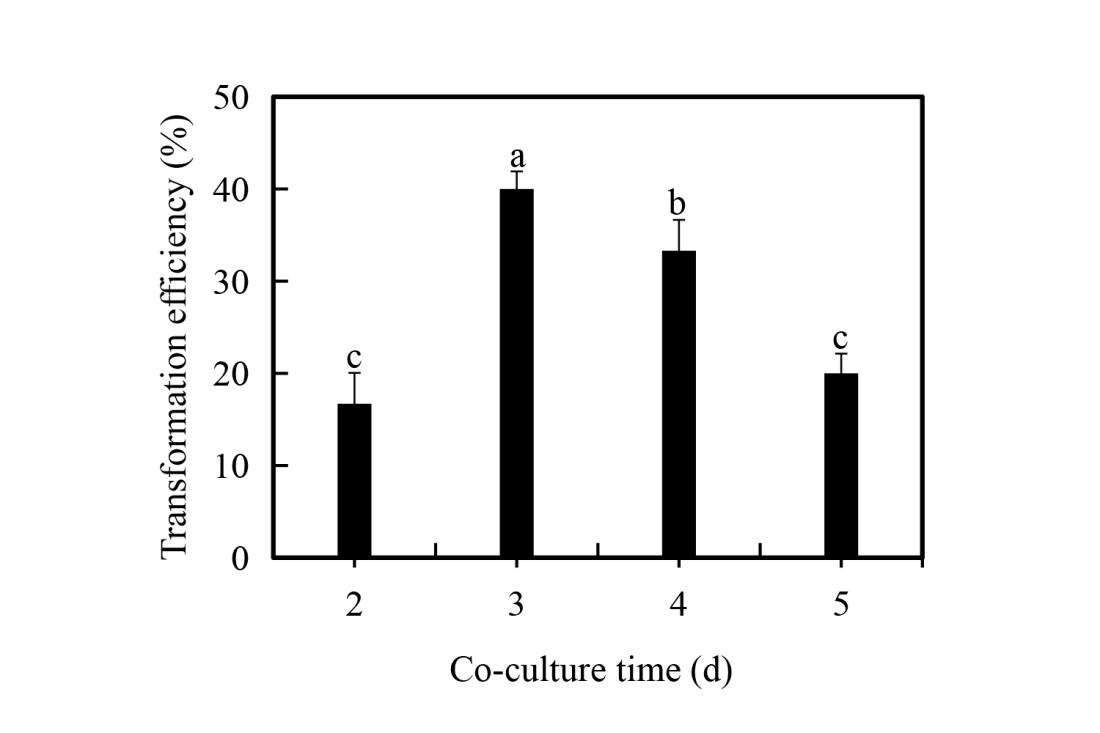


**Supplementary Figure 6.** Effects of co-culture time on transient transformation rate of *P. lactiflora*. Overnight culture of *Agrobacterium* (1.2 OD_600_) was re-suspended in transformation solution (MS + 1 mmol·L^-1^ MES + 2 mmol·L^-1^ MgCl_2_ +200 μmol·L^-1^ AS + 30 g·L^-1^ sucrose, pH=5.6). The germination time was 30, and infection time was 24 h. Error bars indicate standard deviation of three replicates. Different letters mean significantly different by Duncan’s multiple range test at *P* < 0.05.


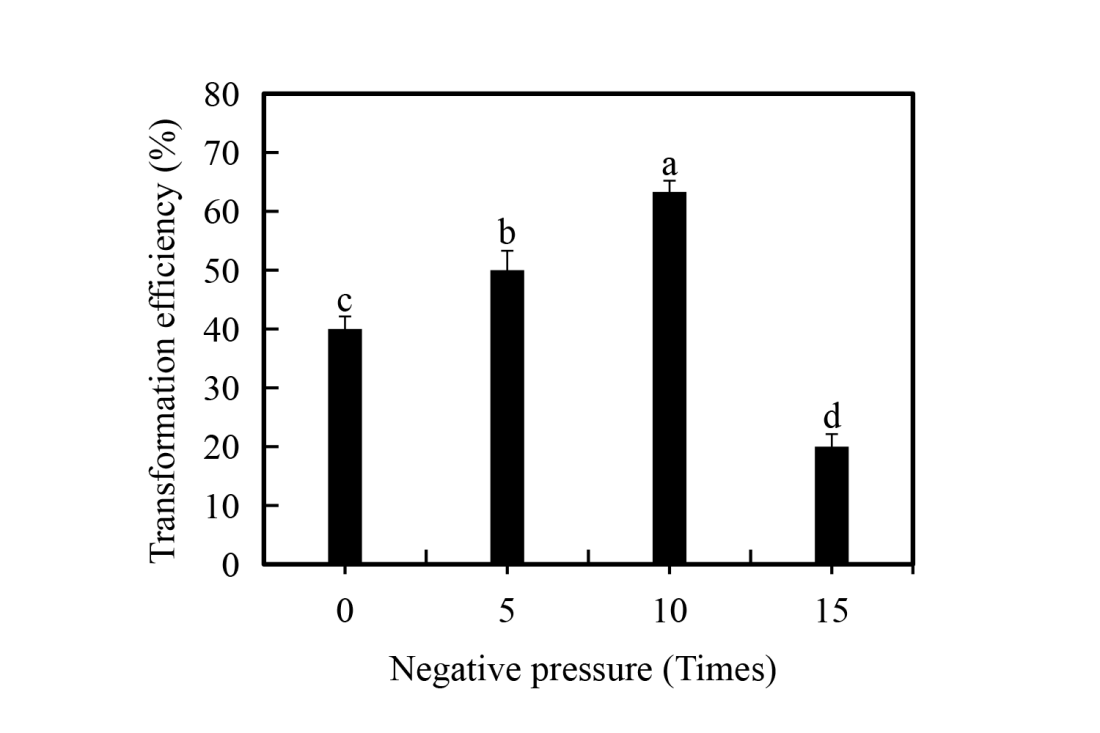


**Supplementary Figure 7.** Effects of negative pressure on transient transformation rate of *P. lactiflora*. Overnight culture of *Agrobacterium* (1.2 OD_600_) was re-suspended in transformation solution (MS + 1 mmol·L^-1^ MES + 2 mmol·L^-1^ MgCl_2_ +200 μmol·L^-1^ AS + 30 g·L^-1^ sucrose, pH=5.6). The germination time was 30, infection time was 24 h, and co-culture time was 3 d. Error bars indicate standard deviation of three replicates. Different letters mean significantly different by Duncan’s multiple range test at *P* < 0.05.

**
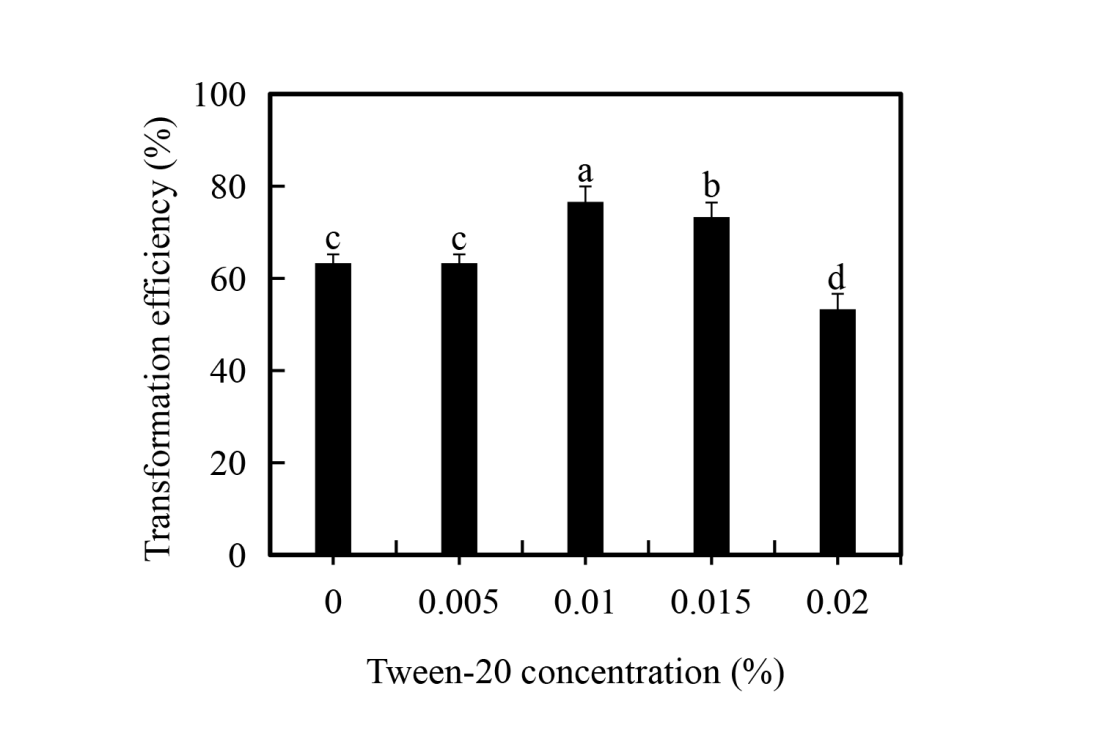
**

**Supplementary Figure 8.** Effects of Tween-20 concentration on transient transformation rate of *P. lactiflora*. Overnight culture of *Agrobacterium* (1.2 OD_600_) was re-suspended in transformation solution (MS + 1 mmol·L^-1^ MES + 2 mmol·L^-1^ MgCl_2_ +200 μmol·L^-1^ AS + 30 g·L^-1^ sucrose, pH=5.6). The germination time was 30, infection time was 24 h, co-culture time was 3 d, and negative pressure was 10. Error bars indicate standard deviation of three replicates. Different letters mean significantly different by Duncan’s multiple range test at *P* < 0.05.





**Supplementary Figure 9.** Effects of infection time on transient transformation rate of *P. lactiflora*. Overnight culture of *Agrobacterium* (1.2 OD_600_) was re-suspended in transformation solution (MS + 1 mmol·L^-1^ MES + 2 mmol·L^-1^ MgCl_2_ +200 μmol·L^-1^ AS + 30 g·L^-1^ sucrose + 0.01% Tween-20, pH=5.6). The germination time was 30, co-culture time was 3 d, and negative pressure was 10. Error bars indicate standard deviation of three replicates. Error bars indicate standard deviation of three replicates. Different letters mean significantly different by Duncan’s multiple range test at *P* < 0.05.

## Supplementary Tables

**Supplementary Table 1 Primer information for PCR**

| Primer name | Primer sequence (5'-3') |
| --- | --- |
| *GUS*-F | GTCGCGCAAGACTGTAACCA |
| *GUS*-R | TGGTTAATCAGGAACTGTTG |
| *PlGPAT*-F | CATGCCATGGCATGATGTTGCTATTCTCTTCTTCGCTTT |
| *PlGPAT*-R | GAAGATCTTCCTAAACCCACGGTTGTGACAAA |
| *PlDHN2*-F | CATGCCATGGCATGATGTCGTACCAAAACCAATATGCAACC |
| *PlDHN2*-R | GAAGATCTTCGTGGTGTCCAGCATGGAGCTTGTCC |
| *PlHD-Zip*-F | CATGCCATGGCATGATGGCAGGTGGGAGAGTCTATAAC |
| *PlHD-Zip*-R | GAAGATCTTCGAACCAAAAGGGTTGATCTTCAACAAC |

**Supplementary Table 2 Primer information for quantitative real-time PCR**

| Primer name | Primer sequence (5'-3') |
| --- | --- |
| *PlGPAT-*F | AAATTGGAGAAAAGCGAGTGA |
| *PlGPAT-*R | GACAAAGAGACGGCTGGAGTA |
| *PlDHN2-*F | ATGTCGTACCAAAACCA |
| *PlDHN2-*R | ATGCTCCTGCTTCTGAT |
| *PlHD-Zip-*F | AAGCCTGCTATGATACCCTC |
| *PlHD-Zip-*R | ACAAGGTTCAGAATTTGCACT |
| *Actin-*F | GGTCTATTCTTGCTTCCCTC |
| *Actin-*R | CCCTCTGCGTCTACACTTTC |
